# Modelling the longitudinal measurement of chronic obstructive pulmonary disease outpatient follow-up in the northwestern Ethiopia

**DOI:** 10.1038/s41598-023-48945-1

**Published:** 2023-12-06

**Authors:** Yoseph Kassa, Habtamu Geremew, Chalachew Gashu

**Affiliations:** 1 Department of Statistics, College of Natural and Computational Science, Oda Bultum University, Chiro, Ethiopia; 2 Department of Nursing College of Health Science, Oda Bultum University, Chiro, Ethiopia

**Keywords:** Medical research, Risk factors, Mathematics and computing

## Abstract

Chronic obstructive pulmonary disease is a condition which can be prevented and treated and is characterized by difficulty of breathing that is not entirely curable. The overall objective of this study was to model the variation of longitudinal measurement over time for outpatients with chronic obstructive pulmonary diseases at the University of Gondar referral hospital. From February 1, 2019, to February 1, 2022, a retrospective study of outpatients with chronic obstructive pulmonary disease was conducted in a hospital. The data was extracted from all patients' data records from the patient’s chart. The information includes the fundamental demographic and clinical details of each outpatients with chronic obstructive pulmonary disease. Mixed linear model were used to investigate the determinant factor of chronic obstructive pulmonary disease. From a total of 266 outpatients, Averages of the ratio of forced expiratory volume to forced vital capacity among chronic obstructive pulmonary disease patients were 0.65, with a standard deviation of 0.043. Comorbidities (average = 2.18, 95% CI 0.43:3.9, P = 0.0133), HIV(average = 4.83, 95% CI 1.94:7.72, P = 0.0012), education (average = 2.98; 95% CI 0.75:4.8, P = 0.008), and weight (average = 0.178, 95% CI 0.045:0.311, P = 0.009) are risk factors for change in forced vital capacity. This study clearly shows that there is a high COPD prevalence in Ethiopia. The risk factors for chronic obstructive pulmonary diseases are the smoking status, comorbidities, HIV, education status of the patient, weight, and time of the visit.

## Introduction

In the world, there are several obstacles, including disease, hunger, poverty, and warfare. However, one of the most enormous challenges today is chronic diseases such as COPD. Chronic obstructive pulmonary ailment is a preventable and treatable disease characterized by airflow hassle that is not fully reversible^1^. Globally, the community is challenged with a number of airflow problem that are typically contemporary and are associated with an unusual inflammatory reaction of the lungs to noxious particles or gases. In short, it is preventable, treatable, airflow obstruction that is not reversible chronic, infection, lung/pulmonary, and exacerbation. Chronic Obstructive Pulmonary diseases (COPD) is the third leading motive of dying worldwide, causing 3.23 million deaths in 2019^2, 3^.

Globally, the most commonly encountered aspect of COPD is tobacco smoking. Other types of tobacco and marijuana are also risk factors for COPD. Outside, occupational, and indoor air pollutants, the latter as a result of the burning of biomass fuels, are the principal COPD risk factors^4^.

Approximately, 60.3% (79/131) of the contributors with COPD provided with cough as the primary respiration symptom. Approximately 55.5% (39/70) of men COPD patients and 65.6% (40/61) of women COPD patients had cough. Breathing signs of cough, mucus, wheezing and shortness of breath are significantly more common in patients with COPD than in people who no longer have COPD^5^.

The amount of air that can be forcedly discharged from our lungs after taking a deep inner breath is referred to as our forced vital capacity (FVC). It is tested by spirometry, a typical respiratory test used to check lung function. COPD has a first-rate impact on public health, particularly due to its increasing occurrence, morbidity, and mortality. COPD is a critical lung illness that, over time, makes it difficult to breathe. COPD is also called other terms, such as emphysema or chronic bronchitis^6^.

Pulmonary rehabilitation is recommended at all stages of COPD. It is a personalized treatment plan that may include therapies such as exercise training, education, and behavioral changes. Its goal is to improve a patient's physical and psychological function^7^.

A small group of researchers determined the prevalence of chronic obstructive pulmonary disorder in Sub-Saharan Africa^8^. However, COPD has turned out to be a growing health problem in sub-Saharan Africa because of tobacco smoking and publicity about biomass fuels. Ninety percent of farm households such as most Sub-Saharan African countries rely on biomass fuel for cooking and heating, which affects both children (acute lower respiratory infections) andwomen (COPD). This is the source of high mortality and morbidity levels in the nearby region^9^. According to a few Ethiopian studies, the prevalence of COPD among adults in Ethiopia is high^10^.

The observer discussed above shows that an up-to-date assessment of the evidence concerning chronic obstructive pulmonary disease in Ethiopia is very low in documentation at the country level. In fact, chronic obstructive pulmonary disease is increasing from year to year, and affects mostly developing countries such as Ethiopia. This implies that additional research is required to identify and evaluate factors or prevalent methods for the change in longitudinal measurement in chronic obstructive pulmonary patients under follow-up.

An investigator study was conducted based on a cross-sectional study design. This study design does not necessarily show the prevalence of disease over time. They used logistic regression to identify determinant factors without considering the correlations within the multiple outcomes or subject-specific random effects. In this study, such problem was solved by considering the correlated and missing data in the longitudinal model. The purpose of this study was to evaluate the disease response to treatment over time and to identify factors that affect change in longitudinal measurement follow up among chronic obstructive pulmonary outpatients. The major objective of this study was to model the variation in forced expiratory volume in second/forced vital capacity over time for outpatients with chronic obstructive pulmonary diseases at the University of Gondar referral hospital. The results of this study provide direction for individuals involved in therapy, patient care, and support, as well as for those creating efficient COPD care and outpatients.

## Methods

### Study design

All patients had a physician diagnosis of COPD, a FEV1/FVC ratio < 0.7, and regular visits. At baseline & three-month interval up to three years. The clinical and socio-demographic data were extracted from patient charts. The study design was a retrospective cohort study design. All patients with chronic obstructive pulmonary disease who were included as outpatients from February 1, 2019, to February 1, 2022, chronic obstructive pulmonary outpatients who had two or more visits and patients whose longitudinal measurement were measured during the follow-up were included in the study period. Patients who had only one piece of information were excluded from this study. The data were obtained from a secondary data source.

### Patients

The study was conducted at the University of Gondar referral hospital in Gondar, Ethiopia. Gondar is located in Amhara regional state in northern Ethiopia, which is 748 km from Addis Ababa^11^. The study was approved by the College of Natural and Computational Science Ethical Clearance Review Committee (reference number: 02/03/974/10/2022) of the University of Gondar referral hospital, Gondar, Ethiopia.

### Variables

#### Dependent variable of the study

Longitudinal measurement result: The progression of forced expiratory volume in seconds over forced vital capacity is measured every three months. Forced expiratory volume in seconds is measured with a spirometer; it is a continuous variable.

#### Independent variables of the study

The independent variables that were factors in the change in longitudinal measurement among chronic obstructive pulmonary outpatients were visit time, sex, age, residence, weight, marital status, smoking, education, comorbidities, HIV, and occupation.

### Longitudinal data analysis

In this study, longitudinal model analysis was used to determine factors that affect the longitudinal change in the ratio of forced expiratory volume to forced vital capacity among chronic obstructive pulmonary diseases. Longitudinal modelling between specific subject variations in forced vital capacity was performed to understand differences among individuals, and the continuous model inside subject variations was employed to analyze changes over time^12^. Data with repeated measurements conducted on a topic over time are known as longitudinal data^13^. Within-subject variation, which is the variance in measurements within each subject and allows for the study of changes over time, and intersubject variation, which is the difference in data between various individuals, are the two sources of variation for longitudinal data^14^. This study focused on continuous, repeated measurements of forced expiratory volume in second among patients with chronic obstructive pulmonary diseases at the University of Gondar referral hospital.

#### Exploratory data analysis

Exploratory data analysis can serve to discover as much of the information regarding the raw data as possible, and plotting individual curves to carefully determine the data should be performed first before any formal model fitting is carried out^15^. Hence, this study explored the data by using descriptive statistics and a profile plot of FVC over the period of the study and assessed the nature of the data by exploring individuals' profiles and the average evolution.

#### Covariance structure

Measurements taken on the same subject seem more likely to be comparable than those taken on different people. Repetition of measurements results in correlation. The variability between longitudinal measures needs to be correctly modeled for the analysis to be valid. The four most commonly used covariance structures are compound symmetry (CS), unstructured (UN), Toeplitz (TOEP), and first-order autoregressive (AR (1)). Among those covariance structures, we used the smallest value of AIC or BIC for comparing covariance^16^.

#### Random intercept model

The random intercept model allows intercepts to vary suggestively across groups. Specifically, a basic example of a random intercept model that is incorporated in the model fitting has clearly dissimilar parts. Those are the fixed part and the random part; the previous is the mixture of the intercept and the coefficient of the explanatory variable times the independent variable, and the final is the collection of dual random relationships^17^.

#### Random intercept and slope model

The intercept andslope in this concept are both random. The basic random intercepts and slopes model was considered.

### Model selection

It is necessary to compare many models using various methodologies and methods in order to choose the most parsimonious model that best fits the provided data. Consequently, comparing various models is crucial for making statistical inferences^18^. The three most widely used model evaluation techniques are the Bayesian information criterion (BIC), likelihood ratio test (LRT), and Akaike's information criterion (AIC). With the small values of AIC and BIC, a better model is needed to fit the data^19^.

### Goodness of fit test

A linear mixed effects model makes the assumption that random effects and residuals are normally distributed and uncorrelated with the error term, and any deviation from this assumption has an impact on parameter estimates and residual effect standard errors. Visually examining residual plots can be utilized to determine whether these effects are typical and to spot any effect categories that are outliers. Taking a closer look at the plot of the fitted values by any covariate of interest versus the standardized residuals will help^20^. The normal quantile plot of residuals by variables was used to determine whether the within-group error assumption of normality was valid.

### Treatment for missing data

Missing values are a frequent problem in many real-world data settings. Various methods of imputed missing information are used in longitudinal studies. Multiple imputations are the most common imputation technique for addressing missing values^21^. There are several factors that result in missing data, including study participant dropouts, measurement errors, and unanswered survey questions. Each missing item is replaced by two or more acceptable values as part of the multiple imputations, which reflect a range of potential values. The method's main benefit is that a data set that may be used to impute has already been produced.

### Statistical analysis

The statistical analysis was carried out with the help of the statistical software packages SPSS version 22 and export to R software. The normality Q-Q plot and histogram were used to check whether the assumptions were false or not. The dependent variable was statistically significantly influenced by variables with a p-value of less than 0.05.

### Ethics approval and consent

Ethical clearance was obtained from the University of Gondar, College of Natural and Computational Science Ethical Clearance Review Committee (reference number: 02/03/974/10/2022), which waived the requirement for informed consent to collect and retrieve the data from patient medical charts. For confidentiality, there were no linkages with individuals and all information had no private identifier and had been kept confidential. Research involving human research participants that performed in accordance with the Declaration of Helsinki.

## Results

The study period was from February 1, 2019, to February 1, 2022, and included 266 chronic obstructive pulmonary outpatients at the University of Gondar referral hospital.

As Table 1 Show that the population consisted of 266 patients with follow-up on COPD drug treatment, of whom 148 (55.6%) were males and 146 (54.9%) lived in urban areas. Approximately 116 (43.6%) patients had a comorbid disease, and 33 (12.4%) COPD patients were HIV positive. Approximately 78 (29.3%) were COPD patients with a smoking status, and regarding marital status, 62 (23.3%), 115 (43.3%), 52 (19.5%), and 37 (13.9%) were single, married, widowed, and divorced, respectively. Regarding their educational status, 108 (347.7%), 86 (32.3%), 36 (13.5%), and 36 (13.5%) patients were uneducated, primary educated, and secondary educated respectively. Approximately 76 (28.6%), 82 (30.8%), 40 (15%), and 68 (25.6%) patients were farmers, merchants, government workers, and others respectively.

**Table 1:** Summary statistics for categorical and continuous variables.

	Categories	N (%)		
Sex	Male	148 (55.6%)		
	Female	118 (44.4%)		
Residence	Rural	120 (45.1%)		
	Urban	146 (54.9%)		
Marital-status	Single	62 (23.3%)		
	Married	115 (43.3%)		
	Widowed	52 (19.5%)		
	Divorce	37 (13.9%)		
Smoking	Non smoker	188 (70.7%)		
	Smoker	78 (29.3%)		
Occupation	Farmer	76 (28.6%)		
	Merchant	82 (30.8%)		
	Government employed	40 (15%)		
	Others	68 (25.6%)		
Education	No educated	108 (40.7%)		
	Primary educated	86 (32.3%)		
	Secondary educated	36(13.5%)		
	Diploma and above	36 (13.5%)		
Comorbidities	No	150 (56.4)		
	Yes	116 (43.6)		
HIV	No	233 (87.6%)		
	Yes	33 (12.4%)		

	Description of continuous variables			
	Min	Max	Mean	Std dev
Age at baseline	18	86	47.03	14.71
Weight at the baseline	41	74	57.28	7.44
FVC	53	94	74.45	8.59
FVE1/FVC	0.51	0.72	0.65	0.043

The averages ratio of forced expiratory volume to forced vital capacity among chronic obstructive pulmonary disease patients was 0.65, with a standard deviation of 0.043. The mean age of chronic obstructive pulmonary disease patients at baseline was 47.03 years, with a standard deviation of 14.71. The mean weight of chronic obstructive pulmonary patients at baseline was 57.28 kg, with a standard deviation of 7.44 kg.

### Distributional study of these data

**Figure 1:**
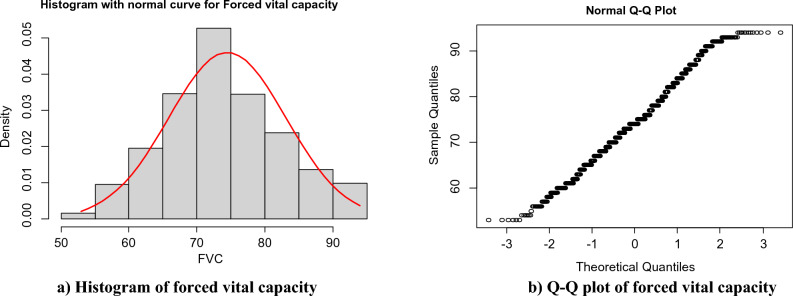
Distributional study of this data.

Figure 1 The histogram and the normal Q-Q plot of forced vital capacity among COPD outpatients showed that the actual data satisfied the assumption of approximately normal.

### Exploratory (profile) data nalysis

**Figure 2:**
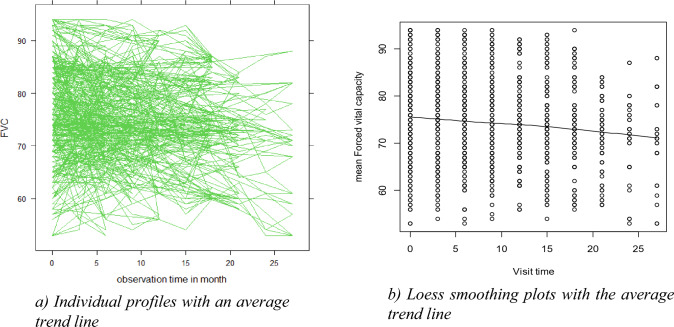
Profile data analysis of COPD.

Figure 2 (a) provides some information on outpatients with chronic obstructive pulmonary disease variability and illustrates that there was a change in forced vital capacity over time. This suggests that patients with COPD outpatients started at different baseline FVC levels, and that patients' evolutions differ at different time points. According to the smoothing plot in (b) which was created using the Loess smoothing approach, the mean structure of the FVC among COPD outpatients is roughly linear over time. (i.e. the relationship between visit time and mean FVC seems to be linear.)

### Exploring the mean structure of FVC for categorical variables

To explore the mean of the ratio of forced expiratory volume in 1 s (FEV1)/vital capacity (FVC) with respect to each categorical variable in the study over observational time, the next plots were considered.

**Figure 3:**
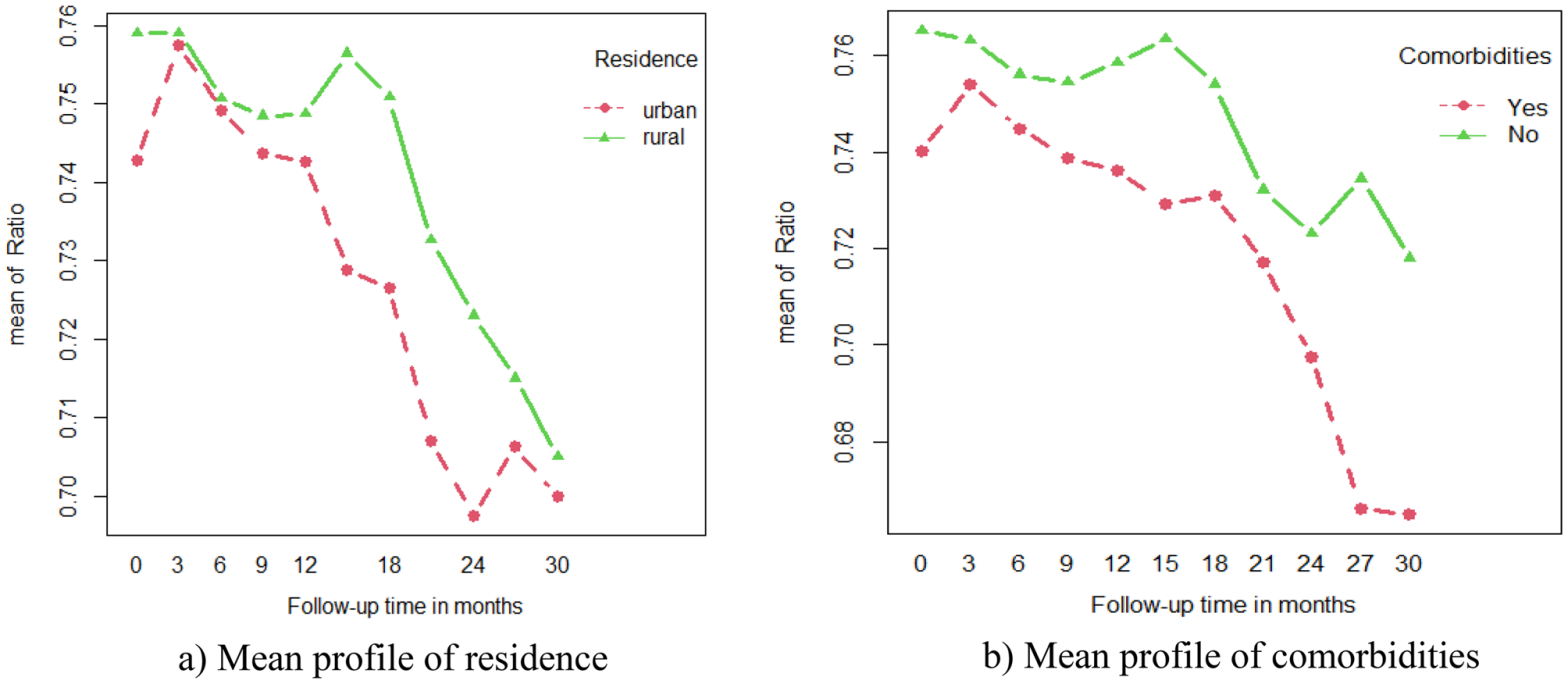
The mean profile plot of Ratio for categorical variables.

Figure 3 displays the mean profile plot of FEV1/FVC by the categorical variables of COPD patients over the follow-up visit time. (a) The mean ratio of COPD patients who lived in rural areas was higher than that of COPD patients who lived in urban areas. (b) The mean ratio of COPD patients with related diseases was lower than that of patients with no related diseases from the start of the baseline to the end of the study.

### Selection of covariance structure

In many clinical trials, repeated measurements made on the same subject were correlated; in this case, this correlation and covariance among the repeated measurements should be modeled accurately. Therefore, a linear mixed effects model with the following most commonly used covariance metrics was fitted, and the one corresponding to the model with the smallest value for fit statistics was selected.

The result from Table 2 revealed that the linear mixed effects model corresponding to the autoregressive first-order covariance structure was the best fit due to its smallest AIC and BIC. This was because the correlation among the repeated measurements on each subject decreased while the distances between visiting times increased. Accordingly the remaining analytical procedures were performed with this structure.

**Table 2:** Selection covariance matrix selection.

Covariance structure	AIC	BIC	Loglik
Unstructured (UN)	10,469.26	10,544.64	− 5220.631
Compound symmetry (CS)	10,471.26	10,552.02	− 5220.631
Autoregressive first order (AR1)	**9899.18**	**9979.94**	** − 4934.59**
Toeplitz (Toep)	9976.85	10,027.24	− 4978.35

Bold values indicate better results than other filtering methods.

## Multivariable analysis of the linear mixed model

The multivariable analysis of the linear mixed effects model was performed by using all the predictor variables that were significant at the 25% level in the univariable analysis. Accordingly, the following table displays the results of the linear mixed effects model (which comprises fixed and random effect estimates of the predictors).

Table 3 Results showed that smoking status, the presence of related diseases (comorbidities), HIV, and weight were positive statistically significant predictors of longitudinal measurement change. However, time visit and education status were negatively associated with longitudinal measurement change among COPD outpatients at a 5% level of significance. Moreover, from the table of random effect estimates, the estimated subject-specific variability was statistically significant at a 95% confidence level. The statistical significance of this parameter supports the assumption of heterogeneous variances for the repeated measurement data.

**Table 3:** Parameter estimates for the linear mixed effects model.

Variables	Categories	Estimate	S.E	95%	CI	p-value
				Lower	Upper	
Estimation for fixed effect						
Intercept		62.282	3.884	54.678	69.843	**0.0000**
Education (ref = no educated)	Primary education	– 2.843	0.996	0.883	4.777	**0.0047**
	Secondary Education	1.887	2.003	– 2.025	5.826	0.3470
	Diploma and above Education	-0.666	1.843	– 4.271	2.954	0.7182
Marital status (ref = single)	Married	– 2.011	1.189	– 4.338	0.319	0.0920
	Widowed	− 1.051	1.376	– 3.755	1.635	0.4455
	Divorce	− 1.311	1.912	– 5.060	2.434	0.4933
Residence (ref = rural)	Urban	0.821	0.943	– 0.950	2.722	0.3849
Sex (ref = female)	Male	0.96	0.931	– 0.858	2.784	0.3033
Comorbidities (ref = no)	yes	2.183	0.876	0.467	3.901	**0.0133**
HIV (ref = no)	yes	1.825	1.476	1.936	7.724	**0.0012**
Smoking (ref = nonsmoker)	smoker	2.790	1.035	0.750	4.802	**0.0075**
Weight		0.178	0.068	0.045	0.311	**0.0091**
Visit time		− 0.153	0.038	− 0.227	− 0.077	**0.0001**

Cov parm	Std. dev (estimate)	95% CI				
		Lower	Upper			
Estimates for random effects						
Intercept (*b*_0*i*_)	7.39	6.677	8.167			
Visit time (*b*_1*i*_)	0.498	0.436	0.568			
Residual	3.99	3.828	4.165			
Corr (*b*_0*i*,_ *b*_1*i*_)	− 0.54					

*ref* reference category of categorical variables, *S.E* standard error. Bold = significance.

The slope and random intercept are correlated, demonstrating that each individual's slope and intercept are correlated with one another. As a result, the correlation between slope and intercept was − 0.54, indicating a negative correlation between intercept and slope of linear time, and the variability within patients was 3.99. As a result, the variability in the intercept between patients was 7.39. The variability in slope between patients was 0.498.

$$\mathrm{ICC}=\frac{{{\widehat{\delta }}_{b0}}^{2}}{{{\widehat{\delta }}_{b0}}^{2}+{{\widehat{\delta }}_{e}}^{2}}=7.39^2 /7.39^2+3.99^2=0.77$$

Seventy-seven percent of the variation in forced vital capacity that is not explained by predictor variables is attributable to outpatients (i.e. outpatients with chronic obstructive pulmonary disease).

Patients’ baseline weight, education status, comorbidities, HIV status, smoking status, and visit time were significantly associated with the longitudinal change in forced expiratory volume in second per forced vital capacity among COPD outpatients. The estimated coefficient of fixed effect of the intercept was 67.54, which indicated that the average of the longitudinal change of forced vital capacity among COPD outpatients was 67.54% by excluding all the variables in the model.

For one more visit, the change in forced vital capacity among COPD outpatients resulted in a 0.15 decrement in the average forced vital capacity, keeping all other variables constant. For a one-unit increase in weight, the average forced vital capacity among COPD outpatients was significantly increased by 6.18%, keeping all other variables constant. COPD outpatients with primary education (OR = 0.058) had a 94.2% lower risk of forced vital capacity than COPD outpatients with no education when all other variables were held constant. COPD outpatients who had a habit of smoking (smoker: OR = 16.2) had a 16 times higher risk than non-smokers, keeping all other variables constant. In addition, patients with HIV-infected (HIV^+^: OR = 3.26) COPD had a 3.26 times higher rate than HIV-uninfected COPD patients, keeping all other variables constant. Furthermore, keeping all other variables constant, COPD outpatients with related diseases (comorbidities, OR = 8.87) had an 8.87 times higher risk than COPD outpatients without related diseases.

## Discussion

In this study the linear mixed effects model for longitudinal change in forced expiratory volume in 1 s was divided by forced vital capacity, and chronic obstructive pulmonary outpatients. In the analysis of longitudinal data, the assumption of normality was checked by histogram and Q-Q plot by comparing the results among COPD patients. All the results indicated that there was an appropriate value. Since the mean of the longitudinal measurement linearly decreasing over time, it was analyzed using the linear mixed effects model by incorporating subject-specific variability. In this study, a linear mixed effects model corresponding to first order autoregressive.

The (AR (1)) covariance structure had smaller AIC and BIC values than the other models. Furthermore, the likelihood ratio test for the random intercept and slope model was statistically significant, and then the linear mixed effects model was fitted with the AR (1) covariance structure for the ratio for FEV_1_/FVC among COPD patients.

The primary finding of our study objective is predictors of longitudinal change in the ratio of forced expiratory volume in one second per forced vital capacity in outpatients with chronic obstructive pulmonary disease. When the change in forced vital capacity level increased by one unit, the weight of patients increased by 0.0618%. This result is also in line with the findings of N. Wang et al. Advanced age (60 years old) was identified as the most important risk factor for COPD (OR 95% CI 3.3). For people aged 40 to 59, smoking was the most important risk factor for COPD (OR 95% CI 2.7). Among people aged 40 to 59, those aged 54 or older with a BMI of less than 23 kg/m2 and a smoking history of more than 33 pack-years had the highest prevalence of COPD (37.5%)^22^.

Our study found that outpatients who were smokers to nonsmokers had higher odds of having chronic obstructive pulmonary diseases. This study was supported by Gashaw G. Woldeamanuel et al.^10^.

The average forced vital capacity of COPD patients who smoke cigarettes was 16 times higher than that of patients who have no habit of smoking cigarettes. This study was supported by Zhang et al. OR 2.55, i.e. the patients who have a smoking habit are 2.55 times more likely to be at risk than patients who have no smoking habit^23^. Also, this study is supported by J. Lee, H. Jung, et al. Almost all patients (98.8%) were smokers compared to the nonsmokers among COPD patients^24^. When the age of patients was increased by one year, the change in forced vital capacity of COPD patients was significantly increased by 0.0928%^23^.

The average forced vital capacity of COPD patients who had related diseases (comorbidities) was significantly higher by 8.87 than the average forced vital capacity of COPD patients who had no related diseases. This finding lines up with the other study by J. M. Figueira-Gonçalves et al. The most frequently associated morbidities were arterial hypertension (59.5%), dyslipidemia (54.3%), and type 2 diabetes mellitus (31.2%); 32% of the patients suffered from heart disease. There is a high prevalence of active smoking, type 2 diabetes mellitus, and heart disease in patients referred for COPD to Canary Island pneumology outpatient services^25^.

Our study has a number of drawbacks. The sample size is initially somewhat small, which could cause some impacts to be either overestimated or underestimated. Therefore, further extensive research is needed to support our findings. Considering the evaluation of a prescreened population’s historical data, the Choice may bias the outcomes. Some outcomes were lacking or insufficient, which could affect the data we have.

## Conclusion

The main goal of this study was to identify the potential risk factors affecting longitudinal measurements in chronic obstructive pulmonary outpatients**.** This study was based on a retrospective study of 266 random samples of chronic obstructive pulmonary patients who were attending their follow-up clinic at the University of Gondar referral hospital in Gondar, Ethiopia, from January 1, 2019, to February 1, 2022.

In this study, the analysis showed that smoking status, education, comorbidities, HIV, observational time, and weight were significantly associated with the change in the quotient of FEV1 to forced vital capacity. This study demonstrates that for estimating the rate at which FEV_1_ to FVC changes over the course of visits for outpatients, a linear mixed effect model with a random intercept and random slope and a first-order autoregressive covariance structure is favored. Regarding time, we discovered that the forced vital capacity pattern showed a linear decline over time, which was further supported by the model that showed a negative estimate of time. According to this study, differences among outpatients accounted for 77% of the variation in forced vital capacity among patients with chronic obstructive pulmonary diseases.

## Data Availability

Ethically, data cannot be deposited in open-access repositories. To obtain the data access, please contact the corresponding author.

## Abbreviations

AIC: Akaike’s information criteria
BIC: Bayesian information criteria
CI: Confidence interval
COPD: Chronic obstructive pulmonary disease
FEV1: Forced expiratory volume in 1 s
FVC: Forced vital capacity
HIV: Human immunodeficiency virus
LMM: Linear mixed model
LRT: Likelihood ratio test
REML: Restricted maximum likelihood
WHO: World Health Organization
